# Whole-Exome Sequencing Revealed a Pathogenic Germline Variant in the Fumarate Hydratase Gene, Leading to the Diagnosis of Hereditary Leiomyomatosis and Renal Cell Cancer

**DOI:** 10.3390/diagnostics14121279

**Published:** 2024-06-17

**Authors:** Akari Nagashima, Sohshi Morimura, Toshihisa Hamada, Takayuki Shiomi, Ichiro Mori, Naoko Sato, Junko Nomoto, Masaki Tanaka, Shoji Tsuji, Makoto Sugaya

**Affiliations:** 1 Department of Dermatology, International University of Health and Welfare, Narita 286-8520, Japan; acr32dligh@gmail.com (A.N.); thamada@iuhw.ac.jp (T.H.); sugayamder@iuhw.ac.jp (M.S.); 2 Department of Pathology, International University of Health and Welfare, Narita 286-8520, Japan; t_shiomi@iuhw.ac.jp (T.S.); ichiro-m@iuhw.ac.jp (I.M.); 3 Center for Genomic Diagnosis, International University of Health and Welfare, Narita 286-8520, Japan; naokosato@iuhw.ac.jp (N.S.); jnomoto@iuhw.ac.jp (J.N.); mtanaka@iuhw.ac.jp (M.T.); stsuji@iuhw.ac.jp (S.T.)

**Keywords:** next-generation sequencing, whole-exome sequencing, multiple leiomyomas, renal cell cancer, uterine myomas, fumarate hydratase

## Abstract

The diagnosis of hereditary skin tumors is difficult for “old” diagnostic tools such as immunohistochemistry. Whole-exome sequencing analysis as a “new” diagnostic tool enables us to make a final diagnosis in spite of unknown hereditary diseases in the past. Hereditary leiomyomatosis and renal cell cancer are autosomal dominant hereditary cancer syndromes characterized by uterine myomas, cutaneous leiomyomas, and aggressive renal cell cancer. The syndrome is associated with pathogenic germline variants in the fumarate hydratase gene. Herein, we demonstrate a pathogenic germline variant of the fumarate hydratase gene in a 60-year-old woman with multiple cutaneous leiomyomas, leading to the diagnosis of hereditary leiomyomatosis and renal cell cancer. Whole-exome sequencing analysis using genomic DNA extracted from peripheral blood leukocytes revealed one germline variant in the FH gene on chromosome 1 (c.290G>A, p.Gly97Asp). She received total hysterectomy due to uterine myoma, which strongly supported the diagnosis. No tumor was detected in her kidney by computed tomography and ultrasound examination. Genetic examination for the mutation of the fumarate hydratase gene is important in order to reach the correct diagnosis and to detect renal cancer at its early stage.

## 1. Introduction

While the diagnosis of genetic skin tumors is difficult for “old” diagnostic tools, whole-exome sequencing analysis as a “new” diagnostic tool is useful for us to make a final diagnosis in spite of unknown hereditary diseases in the past. Hereditary leiomyomatosis and renal cell cancer (HLRCC) is an autosomal dominant hereditary cancer syndrome, which has recently been recognized as an aggressive hereditary RCC. HLRCC is characterized by cutaneous and uterine myomas, as well as an increased susceptibility to develop renal cell cancer associated with pathogenic germline variants in the fumarate hydratase (FH) gene. Hereditary multiple cutaneous leiomyomas were first described in one Italian descent family [1]. Another article demonstrated an autosomal dominant hereditary cutaneous leiomyoma with uterine leiomyoma [2], also known as Reed’s syndrome. Recently, several articles have reported renal cancer syndrome with uterine leiomyomas and cutaneous leiomyomas, which was named HLRCC [3]. Genetic analysis has revealed pathogenic germline variants in FH predispose one to HLRCC [4]. Here, we present a germline variant in FH in a case with multiple cutaneous leiomyomas, leading to the diagnosis of HLRCC.

## 2. Case Report

**Figure 1:**
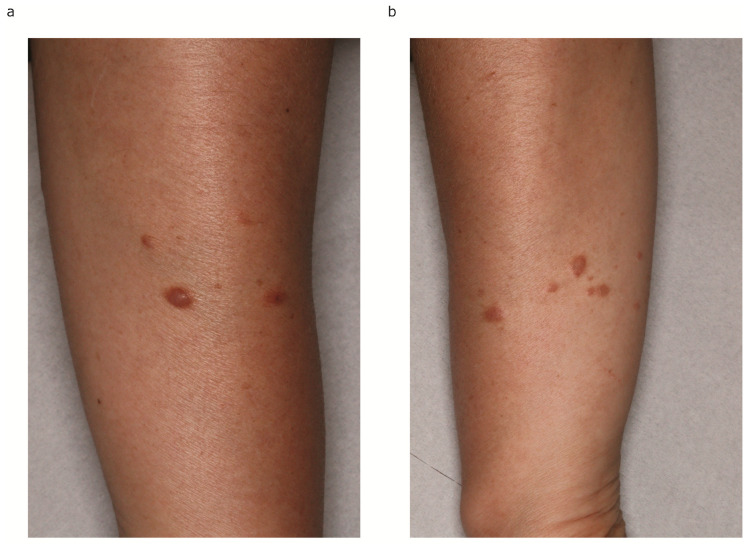
Multiple firm dark-brown papules and nodules ranging from 0.5 to 2 cm in size were arranged on both of her forearms. (**a**) Right forearm. (**b**) Left forearm.

**Figure 2:**
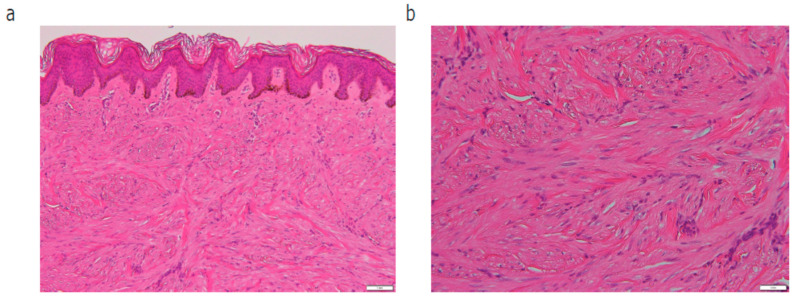
HE staining showing how bundles of spindle cells were increased in the dermis, in (**a**) ×100 and in (**b**) ×400.

**Figure 3:**
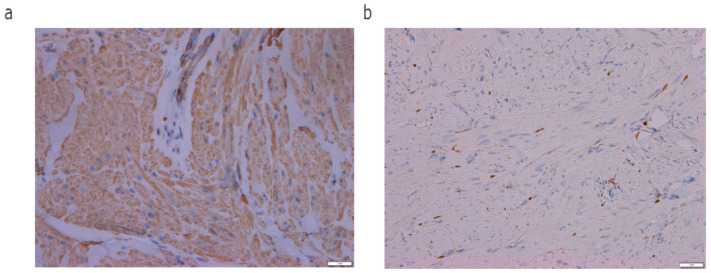
(**a**) Spindle cells in the dermis were positive for α-smooth muscle actin. ×400. (**b**) Spindle cells in the dermis were negative for S-100. ×400.

A 60-year-old Japanese woman admitted to our hospital complaining of more than 10 cutaneous tumors with intermittent pain on both of her arms. The tumors first appeared five years before and gradually increased in number and size in the past two years, becoming more painful to the touch. Multiple firm dark-brown papules and nodules were arranged on both of her forearms (Figure 1a,b). Total hysterectomy was received due to multiple uterine myomas. No family history was found for cutaneous tumor or uterine myoma. In total, four tumors were removed surgically. In a pathological examination, bundles of spindle cells were increased in the dermis (Figure 2a,b). Spindle cells in the dermis were positive for α-smooth muscle actin (α-SMA) (Figure 3a) and negative for S-100 (Figure 3b). All four samples showed a similar histological pattern. Thus, we diagnosed her with multiple cutaneous leiomyomas. A past history of uterine myomas indicated the possibility of HLRCC. Whole-exome sequencing analysis of genomic DNA extracted from peripheral blood leukocytes revealed one germline variant in the FH gene. Taken together, we diagnosed the case as HLRCC. CT scan and ultrasound examination showed no detectable renal tumors. A careful monitoring of developing renal cancer needs to be followed in the future.

## 3. Whole-Exome Sequencing Analysis

Genomic DNA was extracted from peripheral blood leukocytes with written informed consent according to the standard procedure. DNA libraries were prepared using NEBNext Ultra II FS DNA Library Prep Kit for Illumina (New England Biolabs, Ipswich, MA, USA) and xGen Exome Hybridization Panel v2 (Integrated DNA Technologies, Coralville, IA, USA). Whole-exome sequencing analysis was conducted using NovaSeq6000 (Illumina, San Diego, CA, USA). The reads were mapped to GRCh38/hg38 employing BWA-MEM. Variant calling was conducted with GATK4 best practices workflows, including Variant Quality Score Recalibration (VQSR). Based on the clinical information, we searched for variants in FH.

## 4. Results

**Figure 4:**
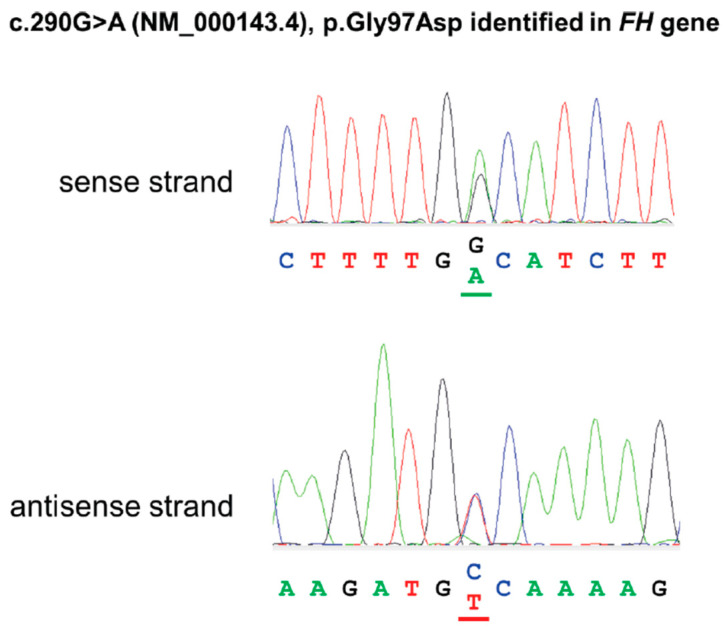
Direct nucleotide sequencing analysis of the PCR products obtained with the primers flanking the variant (c.290G>A (NM_000143.4), p.Gly97Asp identified in FH gene).

Based on the clinical diagnosis, the FH gene was listed as the candidate gene in the exome sequencing analysis. A variant, ENST00000366560.4(NM_000143.4):c.290G>A, NP_000134.2:p.Gly97Asp, was identified as a candidate variant, which replaces the glycine at codon 97 of the FH protein by aspartic acid, an amino acid with dissimilar properties. (Figure 4). This variant was previously reported in a French case of hereditary leiomyomatosis and renal cell carcinoma syndrome [5] (PS1). This amino acid position is highly conserved in vertebrate species. This alteration is predicted to be deleterious by multiple lines of functional prediction (PP3). The FH enzyme activity of the patient was decreased to 40%, which is well below the 78% required for a mutation to be considered deleterious [5] (PS3). This variant is not registered in the Genome Aggregation Database (gnomAD) (PM2). Taken together, this variant was interpreted as pathogenic.

## 5. Discussion

According to the diagnostic criteria [6], HLRCC is definitely diagnosed by detection of pathogenic germline FH variants. The major criteria for a clinical diagnosis of HLRCC are multiple cutaneous leiomyomas with at least one histologically confirmed lesion [6]. Suspicious criteria for HLRCC are solitary cutaneous leiomyoma and a family history of HLRCC, as well as early-onset renal tumors and uterine leiomyomas [6]. Therefore, we diagnosed her with HLRCC due to the FH variant. CT scan and ultrasound examination did not see any evidence of renal cancers. Since HLRCC-associated renal cancers can be more aggressive and provide poorer prognosis than other hereditary renal cancer syndromes, annual image screening is going to be performed in the future.

HLRCC is associated with multiple cutaneous leiomyomas, early-onset uterine leiomyomas, and aggressive RCC. HLRCC-associated RCC tends to be found at a younger age, with a median detection age of 44 years [7]. HLRCC patients with stage III cancer show the worst progression-free survival and overall survival rates [8]. It has been reported that HLRCC patients were confirmed in 8 of 11 patients with multiple piloleiomyomas (73%) [9], which was highly frequent. Patients with leiomyomas without HLRCC were older than patients with HLRCC (56 vs. 34 years, *p* = 0.009) [9]. Another report demonstrated that patients with sporadic leiomyoma were diagnosed with uterine leiomyomas at an older age compared with HLRCC (mean 45 years vs. 33 years, *p* < 0.0001) [10]. Furthermore, patients in the sporadic leiomyoma group underwent surgical treatment at older ages compared with HLRCC (mean 48 years vs. 37 years, *p* < 0.0001) [10]. In our case, the patient was diagnosed with uterine myomas in her twenties and received total hysterectomy around 30 years of age, which is consistent with the characteristics of patients with HLRCC.

A recent study demonstrated that S-(2-succino)-cysteine (2SC) was enhanced in HLRCC-related RCC by immunohistochemistry [11]. Another article reported that two tumor-derived metabolites, succinyl-adenosine and succinic-cysteine, were good plasma biomarkers for the early diagnosis of HLRCC [12]. In addition, it was reported that HLRCC leiomyomas had a dense microvasculature enhanced by CD34 immunostaining when compared with the sporadic leiomyoma group [10]. Furthermore, HLRCC leiomyomas had stronger antiapoptotic protein Bcl-2 immunostaining when compared with the sporadic leiomyoma group [10]. Discovering early diagnostic biomarkers or immunostaining would improve the clinical outcome of patients.

The mechanism of development of RCC induced by FH mutation is still unclear. Recently, it has been reported that FH deficiency induces changes in oxidative carbon metabolism, leading to a switch to aerobic glycolysis and the upregulating of several pro-survival pathways [13]. Pathogenic variants in FH also change tumor cell migratory ability, responding to oxidative stress and reacting to DNA damage [13]. Other studies have demonstrated that FH deficiency accumulates fumarate, which inhibits PTEN to activate PI3K/AKT signaling, resulting in tumor growth [14]. HLRCC tumors overexpress HIF1alpha and hypoxia pathway genes, which result from germline FH mutations [15]. As a limitation, gene examination is not always an easy way for diagnosis due to the cost of medical health insurance.

In conclusion, whole-exome sequencing analysis as a “new” diagnostic tool led us to the diagnosis of HLRCC by detecting a pathogenic germline variant of FH in multiple cutaneous leiomyomas. Genetic examination is essential to achieve a correct diagnosis and to detect aggressive RCC at its early stages.

A part of this abstract was described in Japanese in “Hifuka”.

## Data Availability

Data are contained within the article.
